# Curated gene expression dataset of differentiating 3T3-L1 adipocytes under pharmacological and genetic perturbations

**DOI:** 10.1080/21623945.2020.1829852

**Published:** 2020-10-04

**Authors:** Mahmoud Ahmed, Do Sik Min, Deok Ryong Kim

**Affiliations:** a Department of Biochemistry and Convergence Medical Sciences and Institute of Health Sciences, Gyeongsang National University School of Medicine, Jinju, Republic of Korea; b College of Pharmacy, Yonsei University, Incheon, Republic of Korea

**Keywords:** Gene-expression, microarrays, adipocyte-differentiation, genetic-perturbation, pharmacological-perturbation

## Abstract

The 3T3-L1 cell line is used as an adipocyte differentiation model for the analysis of genes specifically expressed during the differentiation course. This cell model has several applications in obesity and insulin resistance research. We built a data resource to model gene expression of differentiating and mature adipocytes in response to several drugs and gene manipulations. We surveyed the literature survey for microarray datasets of differentiating 3T3-L1 cell line sampled at one or more time points under genetic or pharmacological perturbations. Data and metadata were obtained from the gene expression omnibus. The metadata were manually curated using unified language across the studies. Probe intensities were mapped and collapsed to genes using a reproducible pipeline. Samples were classified into none, genetically or pharmacologically modified. In addition to the clean datasets, two aggregated sets were further homogenized for illustration purposes. The curated datasets are available as an R/Bioconductor experimental data package curatedAdipoArray. The package documents the source code of the data collection, curation and processing. Finally, we used a subset of the data to effectively remove batch effects and reproduce biological observations.

**Database URL**

https://bioconductor.org/packages/curatedAdipoArray

## Background & summary

Adipocytes specialize in storing lipids and triglycerides, therefore they are the primary focus in obesity and diabetes research. To study adipocytes in vitro, researchers leverage the potential of pre-adipocytes such as 3T3-L1 to differentiate into adipocytes by chemical induction [1]. This cell model was successfully used to study lipid synthesis, white vs brown adipose tissue development, insulin-sensitizing drug action [2–4]. Pre-adipocytes execute a well-defined transcriptional programme upon induction that culminates in the accumulation of lipids droplets [1,5]. Transcription factors and co-factors drive the adipocyte differentiation process by inducing the expression of several genes necessary to not only control adipogenesis but also the lipid synthesis and storage. Chromatin immunoprecipitation followed by sequencing (ChIP-Seq) helps to identify the role of transcription factors in the process. Similarly, profiling by microarrays and RNA sequencing (RNA-Seq) facilitates studying the changes in gene expression during the development of the adipocyte phenotype. Over the years, datasets of varying sizes have been generated using different protocols and platforms.

Data repositories such as gene expression omnibus (GEO) and sequence read archive (SRA) provide data in raw and processed forms [6,7]. The diversity of these datasets hinders their reuse by other researchers as it requires significant investment in collecting and processing data. Compiling resources dedicated to the study of particular models may alleviate these difficulties [8]. Careful curation of the metadata and processing the data in standard pipelines increase the utility of the available data. This would allow combining, comparing and integrating data from different studies or types of data. We previously curated two large RNA- and ChIP-Seq datasets for modelling the transcriptional programme of the differentiating adipocyte [9]. Here, we surveyed the literature for microarrays studies using the 3T3-L1 model to study the effect of specific genetic perturbations and chemical compounds on the differentiation course and the behaviour of mature adipocytes. Relevant metadata were manually curated using unified language across the studies, and processed data were made available in a user-friendly format.

In this article, we document the curation process and provide a descriptive analysis of a subset of the data for technical validation. We first describe the induction and perturbation of the pre-adipocyte cell line model. Then, we state the search strategy and the inclusion criteria of the studies. Next, we present the steps and the tools for obtaining and processing the datasets. Finally, using a subset of the data, we perform an analysis for technical validation. We used dimension reduction to evaluate the removal of batch effects and to categorize samples into biologically meaningful groups. We also studied the expression of known adipogenic markers and their expected patterns under peroxisome proliferator-activated receptor gamma (*Pparg*)-knockdown. Together, the documentation and the descriptive analysis provide an assessment for the validity of the model and the appropriateness of the curation process.

## Materials & methods

### 3T3-L1 differentiation & perturbation

3T3-L1 pre-adipocytes differentiate into mature adipocytes when induced with special chemical cocktails [1]. Most studies used a variant containing 1-Methyl-3-isobutylxanthine, Dexamethasone and Insulin (MDI). Fully confluent pre-adipocytes cultures were treated with MDI at day 0, and the media was supplemented with insulin at two-day intervals. Lipid accumulation indicates the progress of differentiation which is usually completed after day 7. The cell cultures were perturbed either before or after the treatment of MDI. Chemical perturbation included synthetic drugs or natural compounds. Genetic perturbation involved the knockdown or the over-expression of key genes. Cell cultures were sampled at one or more time points to isolate RNA for hybridization.

### Data collection & acquisition

We surveyed the literature for ‘3T3-L1’ ‘microarray’ studies. The inclusion criteria were MDI-induced, 3T3-L1, microarray studies with, or without perturbation. Forty-seven public datasets from 43 published studies were found. Datasets and related articles were manually examined for complete metadata and annotations. Six datasets were excluded for missing probe intensities or annotations. The remaining 41 public datasets were retrieved from GEO using GEOquery [10]. A metadata table was created for each study to hold the study and sample-level information. These included: public repository identifiers; differentiation status and time; treatment type, target, does and duration.

### Data processing & packaging

**Figure 1.**
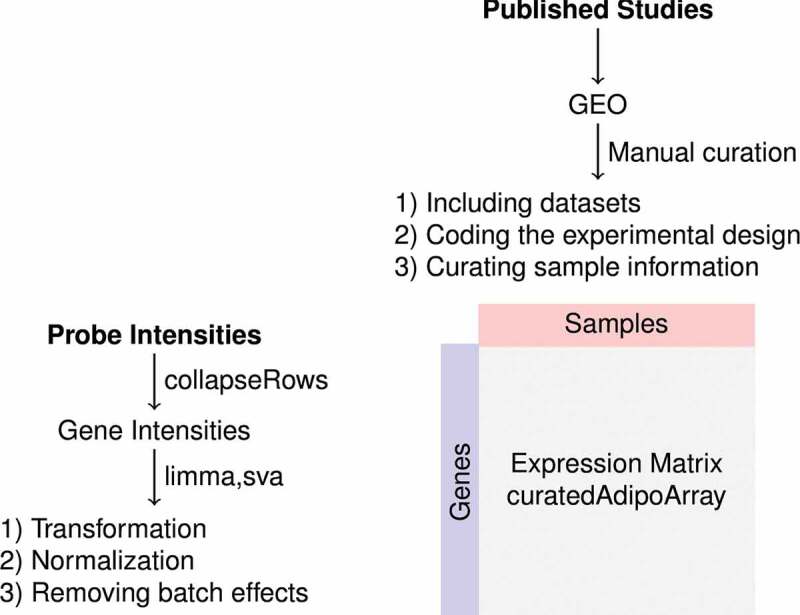
Diagram of the data curation and processing. Metadata were obtained from gene expression omnibus (GEO) and the original articles. Studies were examined and filtered for the details of complete experimental design. Sample information was curated using a unified language across studies. Probe intensities were obtained from GEO, mapped and collapsed to genes. Expression data were log_2_-transformed and normalized, and batch effects were removed. The curated metadata and the expression matrix were packaged in a R/Bioconductor experiment data package (curatedAdipoArray)

Probes were mapped to unique gene symbols. When multiple probes were linked to a single gene, the probe intensities were collapsed to the ‘MaxMean’ using collapseRows [11]. The probe intensities and the sample metadata from each dataset were packaged in R/Bioconductor SummarizedExperiment objects separately [12,13]. The objects were merged into two datasets based on the type of perturbations; pharmacological or genetic. Probes with low intensities (<10) or missing data were removed. The intensities were log_2_-transformed and normalized so they would have similar distributions across arrays using limma [14]. Batch (study of origin) removal procedure was applied using parametric empirical Bayes adjustment (sva) [15,16]. The aggregated datasets were packaged into separate SummarizedExperiment objects and distributed as an R/Bioconductor package (curatedAdipoArray). Figure 1 represents a summary of the data collection, curation and processing.

### Methods of technical validation

Generating *Pparg*-knockdown adipocytes

To illustrate the utility of the curated dataset, we used data from two of the curated studies to show they reflect true adipocyte biology and could be used to extract relevant knowledge after removal of batch effects. The two datasets were constructed by knocking down the peroxisome proliferator-activated receptor gamma (*Pparg*) in the 3T3-L1 pre-adipocytes [17,18]. Cells were transfected with *Pparg* siRNA using lipofectamine or electroporation. Transfected cells were subjected to the standard adipocyte differentiation protocol with MDI as described above. Mature adipocytes were collected around day 7 and hybridized to microarrays.

### Removing batch effects

Data were obtained from two independent studies (GSE12929; n = 48) and (GSE14004; n = 9) [17,18]. Probes were mapped to genes, and the intensities were collapsed, filtered, transformed and normalized as described earlier. The known batch (study of origin) was removed using parametric empirical Bayes adjustment [15]. Dimension reduction using principal component analysis (PCA) was applied before and after the removal of batch effects.

### Differential expression analysis

Gene expression in *Pparg*-knockdown cells (n = 27) was compared to control cells (n = 30) using limma [14]. Fold-change, *p*-value and false discovery rate (FDR) were calculated for each gene. A list of PPARG targets was obtained from [19]. Forty-eight genes were previously identified and experimentally validated as targets for the transcription factor in mouse adipocytes. Twenty-nine of those genes were found in the dataset.

### Gene set enrichment analysis

Differentially expressed genes were ranked based on the fold-change. A list of gene ontology (GO) terms and their mouse gene symbols was prepared using Org.Mm.db.eg and GO.db [20,21]. Gene set enrichment analysis (GSEA) was applied to the list of genes using clusterProfiler [22,23]. Enrichment score (shift towards either end of the ranked list) and *p*-values were calculated for each term to indicate significance. Five relevant GO terms were identified and their results visualized: brown fat cell differentiation (GO:0050873), positive regulation of fat cell differentiation (GO:0045600), response to lipid (GO:0033993), nuclear receptor transcription coactivator activity (GO:0030374) and fatty acid oxidation (GO:0019395).

### Software environment & code availability

The curated datasets are available from Bioconductor experimental hub (https://bioconductor.org/packages/curatedAdipoArray/). The scripts for collecting, processing and packaging the datasets are available on GitHub under GPL-3 licence (https://github.com/MahShaaban/curatedAdipoArray). The code for performing the technical validation is available on (https://github.com/BCMSLab/curated_perturbed_describtor). The software environment where the code was executed is available as a docker image (https://hub.docker.com/r/bcmslab/adiporeg_array).

**Figure 2.**
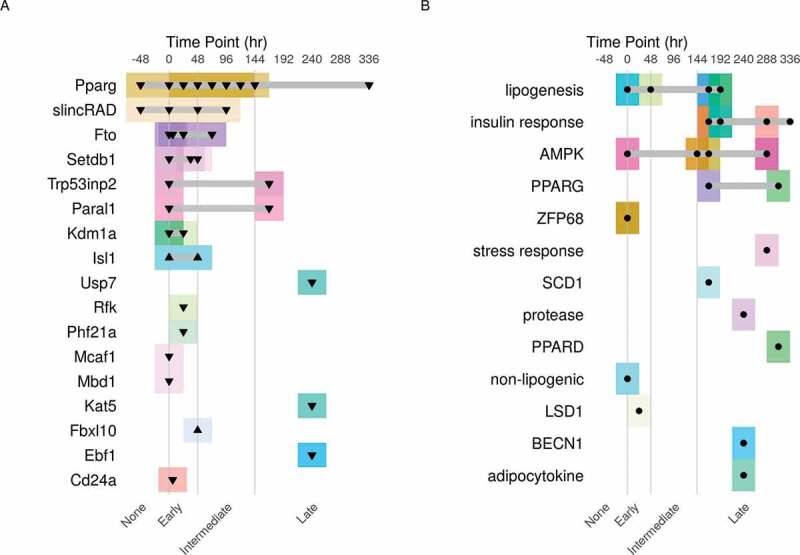
The experimental design of the curated datasets. A schematic description of the experimental design of the MDI-induced 3T3-L1 adipocytes under A) genetic or B) pharmacological perturbations. Each dataset is described by the target of the modification (right), the time point of intervention in hours (top) and the type of modification (▼, knockdown; ▲, overexpression; ● drug treatment). The time course was divided into four stages (0 and before, None; after 0 to 48, Early; after 48 to 144, Intermediate; after 144 hours, Late). Each dataset was represented by a separate colour (box) around the data points

## Results & discussion

### A summary of the data records

We divided the datasets into two main categories based on the experimental design of each study. Figure 2 summarizes the experimental design of the datasets; cell maturation status and the treatment. First, datasets where the differentiation course was modulated using genetic perturbations either through knocking-down or over-expressing genes (Table 1). Second, datasets in which the perturbation was achieved using pharmacological (chemical or natural) compounds (Table 2). In addition, three datasets of gene expression from the same model without perturbations were included for reference [24–26].

**Table 1.** Datasets of genetically modified adipocyte differentiation course. GEO, gene expression omnibus; Ref., reference

GEO ID	Time (hr)	Treatment	Target	Ref.
GSE102403	6	knockdown	Cd24a	[27]
GSE58193	240	knockdown	Ebf1	[28]
GSE64154	48	overexpression	Fbxl10	[29]
GSE69313	0/6/24/72	knockdown	Fto	[30]
GSE40565	48/0	overexpression	Isl1	[31]
GSE28587	240	knockdown	Kat5/Usp7	[32]
GSE25423	0	knockdown	Kdm1a	[33]
GSE18598	24	knockdown	Kdm1a/Rfk	[34]
GSE97241	0/168	knockdown	Paral1	[35]
GSE18599	24	knockdown	Phf21a	[34]
GSE12929	−48/0/24/48/72/96/120/144	knockdown	Pparg	[18]
GSE14004	336	knockdown	Pparg	[17]
GSE73231	0/36/48	knockdown	Setdb1/Mbd1/Mcaf1	[17]
GSE122054	48/96/0/-48	knockdown	slincRAD	[36]
GSE93637	0/168	knockdown	Trp53inp2	[37]

**Table 2.** Datasets of pharmacologically modified adipocyte differentiation course. GEO, gene expression omnibus; Ref., reference

GEO ID	Time (hr)	Target	Treatment	Ref.
GSE42330	240	adipocytokine	asbestose	[38]
GSE14888	144	AMPK	linoleic acide/CLA/metformin/CLA,metformin	[39]
GSE17404	168	AMPK	linoleic acide/CLA/phenoformin	[40]
GSE8681	288	AMPK	linoleic acide/CLA	[41]
GSE8683	0	AMPK	linoleic acide/CLA	[41]
GSE8684	288	AMPK	CLA/linoleic acide	[41]
GSE94539	0/144	AMPK	adenine/AICAR	[42]
GSE56440	240	BECN1	berberine	[43]
GSE1458	288	insulin response	pioglitazone/rosiglitazone/troglitazone	[44]
GSE14810	168	insulin response	rosiglitazone	[45]
GSE24105	168	insulin response	dexamethasone	[46]
GSE4683	192	insulin response	insulin, glucose/rosiglitazone,insulin,glucose/glucose/insulin/rosiglitazone	[47]
GSE56688	168	insulin response	rosiglitazone	[48]
GSE62635	336	insulin response	dexamethasone/TNF	[49]
GSE18598	24	Kdm1a/Rfk/LSD1	siRNA/LSD1 inhibitor	[34]
GSE21157	48	lipogenesis	bilberry/anthocyanidins	[50]
GSE42220	192	lipogenesis	SFA	[51]
GSE53004	0	lipogenesis/non-lipogenic	rosiglitazone/butylparaben/ethylene brassylate/bis (2-ethylhexyl) phthalate/bisphenol A/butylbenzyl phthalate/propylparaben/tributyltin	[52]
GSE26207	312	PPARD/PPARG	PPARD agonist/PPARG agonist	[53]
GSE64075	168	PPARG/insulin response	diclofenac/indomethacin/rosiglitazone/ibuprofen/GQ16	[54]
GSE70854	240	protease	ritonavir/tenofovir/ritonavir,dmso/ritonavir,CLA	[55]
GSE51905	168	SCD1	SCD inhibitor	[56]
GSE8682	288	stress response	tunicamycin	[41]
GSE15018	0	ZFP68	prieurianin	[57]

A transcriptional programme drives the differentiation of adipocytes through a series of factors which induce the expression of genes needed for the metabolic and morphological changes (Table 1 & Figure 2(a)). The most important of these factors is PPARG. By knocking down the gene coding for PPARG in pre-adipocytes, researchers could identify its contribution to the differentiation course. Other aspects of gene regulation such as histone modification were studied by perturbing genes like *Setdb1, Kdm1a, Kat5, LSD1* and *Phf21a*. Genes coding for DNA binding proteins such as *Mbd1* and *Mcaf1* and non-coding genes such as *Paral1* and *slincRAD* were also studied for their role in controlling gene expression.

To study adipocytes *in-vitro*, 3T3-L1 pre-adipocytes are induced to differentiate for 7 to 14 days. Researchers use the mature adipocytes to study the behaviour of fat cells in response to certain stimuli (Table 2 & Figure 2(b)). Adipocytes are specialized in lipid synthesis and storage of excess energy. Several studies used this cell to model insulin responses to drugs such as rosiglitazone, dexamethasone or pioglitazone. All are drugs used to treat diabetes. Similarly, responses to short-chain fatty acids and other lipogenic compounds were investigated. The effect of linoleic acids and metformin on the adipocytes through AMPK was also studied. Finally, the role of adipocytes in creating an inflammatory environment is recognized as harmful and has been studied using asbestos and adipocytokine stimulants.

### Stage of differentiation explains the variance between samples after removing batch effects

**Figure 3.**
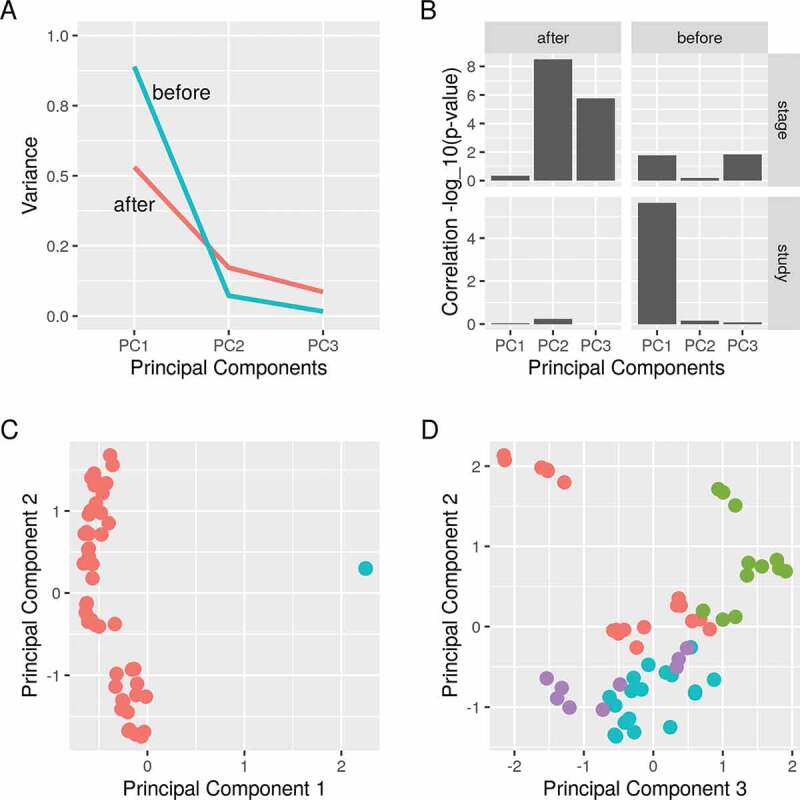
Principal component analysis of differentiating Pparg-knockdown 3T3-L1 pre-adipocytes. Principal component analysis was applied before and after removing batch effects. A) The amount of variance explained and B) the correlation of (study) and (stage) of differentiation with the first three components are shown before and after applying the procedure. C) The first and second principal components are shown. Samples are coloured by the study of origin (*red*, GSE12929; *blue*, GSE14004). D) The second and third principal components are shown. Samples are coloured by the stage of differentiation (*red*, none; *green*, early; *blue*, intermediate; *magenta*, late stage)

The study of origin explained the most variance between the samples before removing the batch effects (Figure 3(a,b)). Using empirical Bayes adjustment, we were able to remove these effects. Afterwards, the stage of differentiation accounted for more variance (Figure 3(a,b)). The first component of the PCA separated the samples before applying the batch removal procedure (Figure 3(c)). Growing adipocytes in an induction media cause substantial changes in the gene expression which is captured in three stages of early, intermediate and late differentiation. Stage of differentiation corresponded to the second and the third components and successfully separated the samples after applying the procedure (Figure 3(d)). Together, removing the study batch reduced the influence of the unwanted variable and gave a meaningful sample separation.

To combine datasets from different sources is to increase the chances of detecting true signals. Independent datasets can be incorporated in a meta-analysis or merged into a larger dataset. One of the main challenges when combining data is the batch effects. It arises from the discrepancies between the datasets such as the cell origin, reagents and platforms on which the data were generated [15]. As a proof of concept, we used empirical Bayes adjustment to maximize the signal derived from two datasets for further analysis [16].

### Pparg*-knockdown results in failure of induction of key target genes*

The adipogenic transcription factor PPARG is the master regulator of the adipocyte differentiation. PPARG regulates the transcription of key adipogenic and lipogenic genes especially in the intermediate-late stage of differentiation [58]. The knockdown of *Pparg* would most probably result in the failure of the differentiation/maturation of the induced 3T3-L1 [59]. We selected a subset of the data where *Pparg* was knocked down in 3T3-L1 pre-adipocytes prior to the MDI-induction. We studied the effect of the knockdown on the differential expression of key targets and gene set enrichment of related-gene ontology terms.

**Figure 4.**
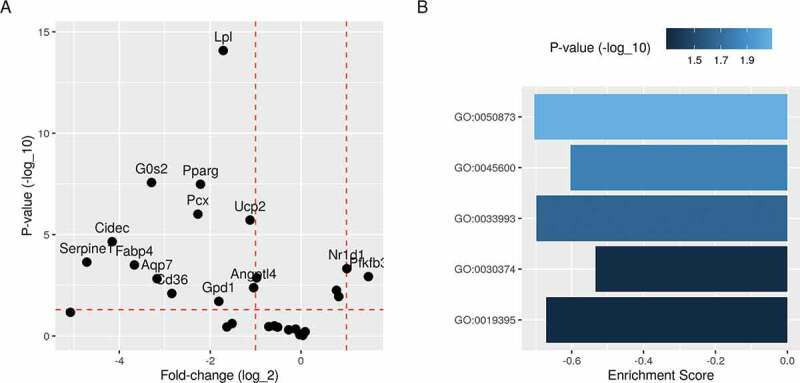
Differential expression and gene set enrichment analysis of differentiating *Pparg*-knockdown 3T3-L1 pre-adipocytes. Differential expression analysis was applied to the process data to identify regulated genes between *Pparg*-knockdown and control conditions. A) Fold-change (log_2_) and *p*-values (-log_10_) of previously identified PPARG target genes are shown. B) Differentially expressed genes were ranked by fold-change and used to calculate the enrichment score and *p*-values of relevant gene ontology terms. Brown fat cell differentiation (GO:0050873); positive regulation of fat cell differentiation (GO:0045600); response to lipid (GO:0033993); fatty acid oxidation (GO:0019395); nuclear receptor transcription coactivator activity (GO:0030374)

The knockdown of *Pparg* in the pre-adipocytes resulted in the failure of induction of key gene targets [19]. Most downstream genes from PPARG were down-regulated in the *Pparg*-knockdown cells compared to the controls (Figure 4(a)). These included key fatty acids metabolism and lipid synthesis genes. Lipoprotein lipase (*Lpl*), cell death-inducing DFFA like effector C (*Cidec*) and *Cd36* were one or more log_2_ fold less in expression than in controls. These genes have essential functions during the differentiation process. Their down-regulation would likely mean that cells would accumulate less lipid and fail to differentiate into mature adipocytes.

### Pre-adipocytes fail to mature under the knockdown of key adipogenesis regulators

The effect of *Pparg*-knockdown can be also evaluated on a gene set level. When the knockdown and control conditions were compared, several key gene ontology terms were significantly enriched (Figure 4(b)). For example, two cell differentiation terms – brown fat cell differentiation (GO:0050873) and positive regulation of fat cell differentiation (GO:0045600) – had higher fractions of their gene members down-regulated by the transcription factor *Pparg* knocking down. Similarly, response to lipid (GO:0033993) and fatty acid oxidation (GO:0019395) were significantly enriched. Finally, nuclear receptor transcription coactivator activity (GO:0030374) is a key molecular function that was enriched during differentiation.

## Concluding remarks

We collected two datasets of previously published microarrays examining differentiation of 3T3-L1 under genetic and pharmacological perturbations. The metadata of the studies were curated and the data were processed and homogenized. We analysed a subset of the data where the key adipogenic transcription factor coding gene (*Pparg*) was knocked down before the induction. We illustrated a method for removing batch effects and generating biologically meaningful results.
